# A functional neuroimaging study of self-other processing alterations in atypical developmental trajectories of psychotic-like experiences

**DOI:** 10.1038/s41598-022-20129-3

**Published:** 2022-09-29

**Authors:** Roxane Assaf, Julien Ouellet, Josiane Bourque, Emmanuel Stip, Marco Leyton, Patricia Conrod, Stéphane Potvin

**Affiliations:** 1 Centre de Recherche, Institut Universitaire en Santé Mentale de Montréal, 7331 Hochelaga, Montreal, H1N 3V2 Canada; 2 Department of Psychiatry and Addiction, Faculty of Medicine, University of Montreal, Montreal, Canada; 3 Centre de Recherche du Centre Hospitalier, Universitaire Sainte-Justine, Montreal, Canada; 4 Department of Psychiatry, Perelman Faculty of Medicine, University of Pennsylvania, Philadelphia, PA USA; 5 Department of Psychiatry, Faculty of Medicine, McGill University, Montreal, Canada

**Keywords:** Neuroscience, Psychosis, Schizophrenia

## Abstract

Self-disturbances constitute a hallmark of psychosis, but it remains unclear whether these alterations are present in at-risk populations, and therefore their role in the development of psychosis has yet to be confirmed. The present study addressed this question by measuring neural correlates of self-other processing in youth belonging to three developmental trajectories of psychotic experiences. Eighty-six youths were recruited from a longitudinal cohort of over 3800 adolescents based on their trajectories of Psychotic-Like Experiences from 12 to 16 years of age. Participants underwent neuroimaging at 17 years of age (mean). A functional neuroimaging task evaluating self- and other-related trait judgments was used to measure whole-brain activation and connectivity. Youth who showed an increasing trajectory displayed hypoactivation of the dorsomedial prefrontal cortex and hypoconnectivity with the cerebellum. By contrast, youth who showed a decreasing trajectory displayed decreased activation of the superior temporal gyrus, the inferior frontal gyrus, and the middle occipital gyrus. These findings suggest that the increasing trajectory is associated with alterations that might erode distinctions between self and other, influencing the emergence of symptoms such as hallucinations. The decreasing trajectory, in comparison, was associated with hypoactivations in areas influencing attention and basic information processing more generally. These alterations might affect the trajectories’ susceptibilities to positive vs. negative symptoms, respectively.

## Introduction

Psychosis risk is understood as existing on a continuum^1^ with, at one end, schizophrenia; at the intermediate, a treatment-seeking Clinical High Risk syndrome characterized by “attenuated” or “brief” symptoms that do not necessarily meet full diagnostic criteria; and, at the lower end, Psychotic-Like Experiences (PLEs). PLEs are defined as subclinical positive psychotic symptoms that can be observed in the general population, including 17% of children aged 9–12 and over 7% of adolescents^2^. While most PLEs are transient in nature and do not extend into adulthood, persistent PLEs confer a four- to ten-fold increased risk for psychosis^3–5^.

Recent cohort studies provide evidence of heterogeneous PLE developmental trajectories during adolescence. In 2566 youths from the general population followed between the ages of 13 and 16, three trajectories were identified: 84% of adolescents (n = 2152) exhibited a typical trajectory of low PLE levels that decreased further over time, 8% (n = 203) exhibited high PLE levels that subsequently decreased, and another 8% (n = 211) reported moderate PLE levels that increased^6^. These trajectories have been replicated in multiple samples^7–9^, supporting their relevance in adolescent development. While the increasing trajectory is thought to be associated with increased risk of psychosis^10^, outcomes in the decreasing trajectory have been little investigated. Our own studies suggest that both trajectories can develop problems, with the increasing trajectory exhibiting progressively more positive psychotic symptoms while the decreasing trajectory displays more negative symptoms; and that both of these atypical trajectories are associated with altered neural profiles during facial emotion processing^11^. That these PLE trajectories can be dissociated from low risk youth and from each other on psychosis relevant clinical and neural measures supports their use in identifying and studying vulnerability to psychotic disorders before the onset of the disease, thereby limiting the confounding effect of antipsychotic medications and comorbidities. This research also suggests potentially two different psychosis profiles with distinct neurodevelopmental markers, but further research is needed to better characterise the neural and information processing differences between these two risk trajectories.

Disturbances in self-reference processing, also called self-disturbances, have been recognized as a core feature of psychotic disorders^12^. These self-disturbances include feelings of derealization, depersonalization, and alterations in the stream of thought^13,14^. They also encompass the alterations in the perception and understanding of the self, the perception and understanding of others (known as theory of mind), and impairments in self-other integration and distinction^15,16^. Furthermore, these alterations are present in the early stages of psychosis^17^ and predict transition to psychosis in at-risk individuals^18,19^. Based on these observations, self-disturbances might constitute a phenotypic marker of psychosis and should be further investigated in clinical and at-risk populations.

Strong evidence shows that information processing relevant to self and other is regulated by cortical midline structures^20,21^. A meta-analysis of functional magnetic resonance imaging studies (fMRI) on self- and other-related judgement tasks showed that self-other processing in healthy volunteers is associated with activations in the medial prefrontal cortex (MPFC), temporoparietal junction (TPJ), and posterior cingulate cortex (PCC)^22^. Similarly, false belief tasks, which aim to test the awareness of others (theory of mind), activate the MPFC, TPJ, precuneus, and middle temporal gyrus^23^. Other frontal areas are also recruited during self-processing, seemingly as part of a wider attentional network^24^.

The self-other processing network overlaps with the Default-Mode-Network in the anterior MPFC, PCC, and TPJ^24,25^. While the Default-Mode-Network was first thought to be a task-negative network only, it is now documented as being also a task-positive network that contributes to different types of tasks^26^. It can be further divided into subsystems that are involved in different aspects of introspective processes^27^: (1) the midline core (comprising the anterior MPFC and PCC), which plays a role in mentalizing and social processing; (2) the dorsal medial prefrontal cortex system (dorsomedial PFC, TPJ, lateral temporal cortex, temporal pole), which is involved in self-referential processes, such as autobiographical memory and self-other distinction; and (3) the medial temporal lobe memory system (ventromedial PFC, posterior inferior parietal lobule, restrospinal cortex, parahippocampal cortex, hippocampus), which plays a role in episodic contextual memory.

Neural alterations in self-processing have been seen with some consistency in people with schizophrenia. A recent meta-analysis of fMRI studies on self-other processing in schizophrenia^28^ identified hypoactivations in the dorsal anterior cingulate cortex (ACC) and the dorsomedial PFC, which are involved in cognitive control, salience attribution, decision-making and self-other differentiation. This suggests an impaired ability to direct attention towards the self in schizophrenia and/or self-other distinction deficits. Although less replicated, some evidence suggests that schizophrenia patients fail to activate the right TPJ, a region influencing self/other differentiations^29^. Furthermore, this study found hyperactivations in the lateral frontal cortex during both self- and other-reflection, suggesting a greater cognitive demand for this task in psychosis^29^. While less explored, it has been shown that schizophrenia patients exhibit reduced functional connectivity between the insula and precuneus/PCC during tasks of other-reflection^30^. This may indicate impaired integration of autobiographical memory and emotional awareness in relation to others.

Only a few studies have investigated neural correlates of self-other processing in at-risk individuals. Ultra-High-Risk (UHR) subjects showed less activation in the ventromedial PFC during self-reflection^31^. Similarly, it has been shown that CHR individuals display decreased connectivity between the MPFC and the PCC compared to controls during a self-reference task, but increased connectivity between those two regions during resting-state^32^. Furthermore, positive schizotypal traits are associated with activation levels of the dorsomedial PFC, the dorsolateral PFC, the PCC and the lingual gyrus during self and other-processing^33^. To date, only one study has evaluated self-other processing in pre-clinical youths with high levels of PLEs. Youths with high psychosis-proneness displayed increased activation in the insula, the dorsomedial PFC, the ventromedial PFC for positive self-related traits, and in the insula, ACC cortex, and dorsomedial PFC for negative self-related traits^34^. It is worth noting that this study only looked at 36 individuals in total.

The current study aimed to investigate the neural correlates of self- and other- related processing in youths belonging to distinct developmental trajectories of PLEs. Since self-disturbances may constitute a better predictor of transition to psychosis than even prodromal symptoms^19^, the current study measured neural correlates of self- and other-related processing in youths belonging to distinct developmental trajectories of PLEs. We hypothesized that the increasing PLE trajectory would display altered activation of brain regions involved in self-other differentiation (dmPFC and TPJ), while our analyses for the decreasing trajectory remained exploratory since its risk profile has not been characterized yet.

## Methods

### Study design and participants

The ongoing Pro-Venture study is a 3-year longitudinal neuroimaging study that follows adolescents who were included in one of three different PLE trajectories over a previous 5-year period during early adolescence. It constitutes a neuroimaging add-on to the larger Co-Venture study^35^. The Co-Venture cohort included 3966 Grade 7 students from 31 high schools of the greater Montreal area who completed annual clinical assessments from 13 to 17 years of age. A growth mixture model was used with the 4-year data (from ages 13 to 16) identified three developmental trajectories of PLEs: a low-decreasing trajectory (control group, PLE-0), a high-decreasing trajectory (decreasing group, PLE-1), and a moderate-increasing trajectory (increasing group, PLE-2)^6^. In brief, group-based trajectories were estimated using growth mixture models which were then fitted with different models ranging from one to four trajectories. The three-trajectory model was determined to be the best-fitting model using the Bayesian Information Criterion, the Akaike Information Criterion, the Lo-Mendell-Rubin Likelihood Ratio Test, and entropy. Missing data on the PLE score were handled through Full Information Maximum Likelihood.

The Pro-Venture sub-cohort consists of 86 adolescents (PLE-0 n = 41; PLE-1 n = 19; PLE-2 n = 26) aged 16–20 at entry (mean age 17.22, SD = 0.72; 52.3% girls). Participants from the three trajectories who had consented to being contacted for future research were identified and were recruited randomly. The results presented here are based on data collected at the first neuroimaging assessment. Ethical approval was obtained from the CHU Sainte-Justine Research Ethics Committee in Montreal and the study and was conducted according to the declaration of Helsinki. All participants actively assented or gave informed consent to the study procedures, and parental informed consent was obtained for minors.

### Exclusion criteria

Exclusion criteria for the Pro-Venture study included DSM-5 psychiatric disorders, family history of schizophrenia, taking antipsychotic medication, neurological disorders or IQ < 70 and a contraindication to undergo MRI examination. A urinary drug screening test was administered to assess recent substance use, with participants testing positive for alcohol, cannabis, MDMA, cocaine or opioids having to postpone their neuroimaging session. Since cannabis metabolites can be detected up to 7 days following use, individuals who tested positive but reported not having consumed cannabis within 24 h and showed no sign of intoxication prior to neuroimaging took part in testing.

### Clinical and behavioral assessments

To develop the adolescent PLE trajectories, past year PLEs (hallucinations, delusional beliefs, suspiciousness, strange experiences, and feelings of grandiosity) were assessed with the *Adolescent Psychosis Screening Scale* using nine items that have previously been described^6,36^. Three of the questionnaires’ items were shown to have significant positive predictive power for interview-verified PLEs that ranged between 80 and 100%^37^. Substance use during the past 12 months was measured using the DEP-ADO questionnaire^38^. Participants rated their consumption of alcohol, tobacco, cannabis and other substances on a 6-level scale ranging from “never” to “everyday”^39^.

We also administered a mentalizing (theory of mind) task adapted from Achim et al., which included 30 stories^40^. In this task, participants were presented with stories containing false beliefs (where one character has a false idea about another character’s beliefs) which served as a measure of second-order mentalizing. This task also contains items assessing non-social reasoning and first-order inference, and items controlling for inattention, memory lapses and false positives. Total scores and the percentage of correct answers were determined.

### Task design

**Figure 1:**
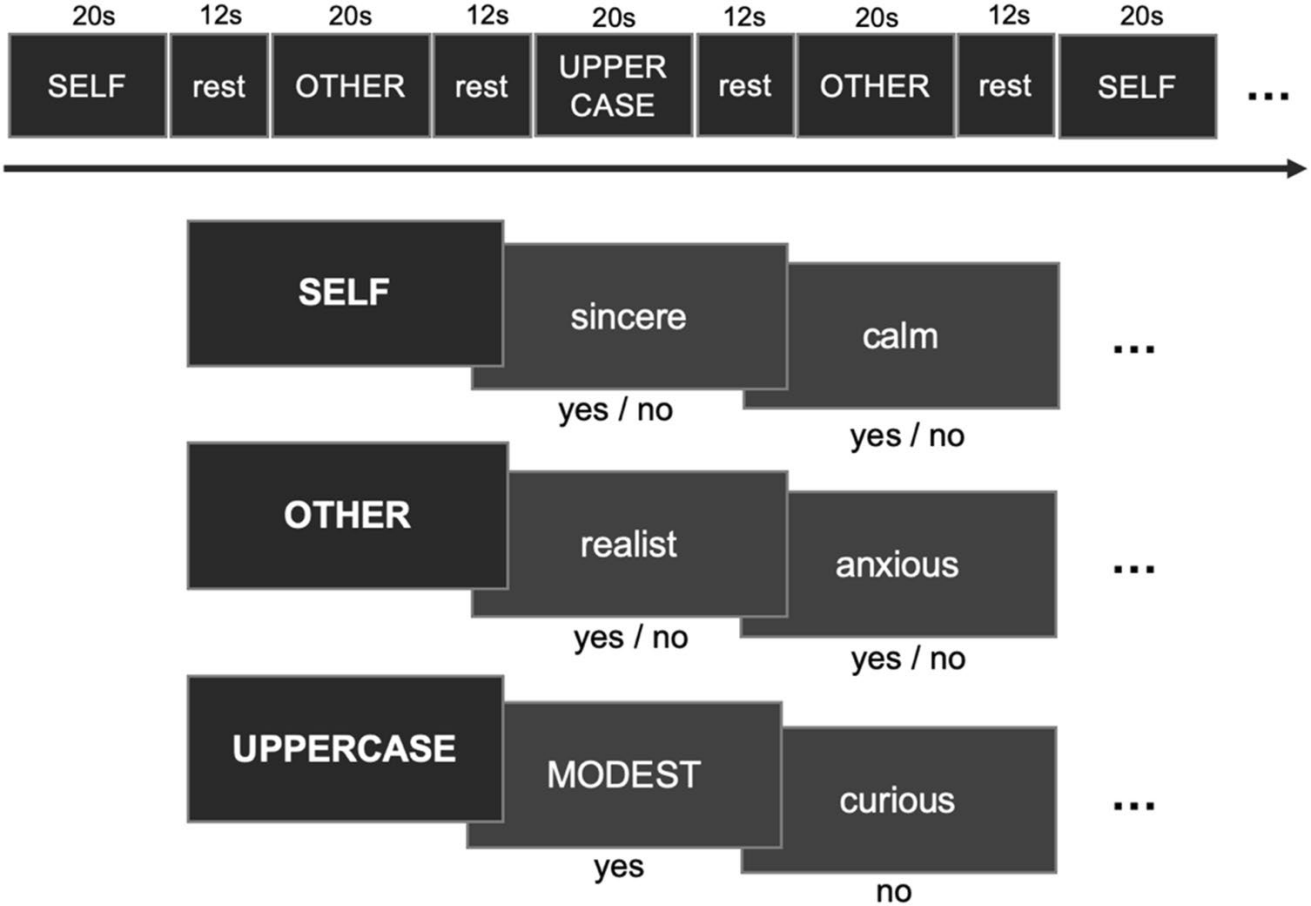
Self-other processing task block-design. The self-other processing task consisted of 15 blocks of 20 s each, interspersed by 12-s blocks of rests. During “Self” blocks, participants were instructed to think about themselves and answer “yes” or “no” to indicate whether the adjective displayed on the screen applies to them or not. During “Other” blocks, participants were instructed to think about one person they know and then answer “yes” or “no” to indicate whether the adjective displayed on the screen applies to that person. During “Uppercase” blocks, which served as a control condition, subjects were instructed to press “yes” if the displayed word was in uppercase, and “no” if it was in lowercase.

We used a self-other processing paradigm based on a block-design that has been successfully employed in several fMRI studies and has consistently shown robust activations in default-mode network brain regions^41^. We selected a total of 75 positive and negative trait adjectives from Anderson’s list of personality traits^42^. The task design is shown in Fig. 1. Three types of blocks were used: five “Self” blocks, five “Other” blocks, and five “Uppercase” blocks. Each block had a duration of 20 s and contained five different adjectives. Blocks were interspersed with a rest period of 12 s. The “Self” and “Other” blocks displayed positive and negative adjectives. During “Self” blocks, participants were instructed to think about themselves and answer with “yes” or “no” to indicate whether the adjective displayed on the screen applied to them or not. During “Other” blocks, participants were instructed to think about one person they know, and then answer “yes” or “no” to indicate whether the displayed adjective applied to that person or not. During “Uppercase” blocks, which served as a control condition, subjects were instructed to press “yes” if the displayed adjective was in uppercase, and “no” if the word was in lowercase. The “Uppercase” block (i.e. control condition) displayed positive and negative adjectives either in uppercase or in lowercase. Participants were given a button box on which they pressed “yes” with their index finger and “no” with their middle finger.

### Neuroimaging acquisition parameters

A 3-Tesla Prisma Fit scanner (*Unité de Neuroimagerie Fonctionnelle de l’Institut de Gériatrie de l’Université de Montréal*) was used to acquire Blood Oxygenated Level Dependent (BOLD) signal. High-resolution structural images were collected with a T1-weighted anatomical images were acquired (TR = 2300 ms; TE = 2.98 ms; FA = 9°; matrix size = 256 × 256; voxel size = 1 mm^3^; 176 slices). In the same session, functional imaging data were acquired using a T2-weighed multiband echo planar imaging sequence (TR = 785 ms; TE = 30 ms; FA = 54°; matrix size 64 × 64, voxel size 3 mm^3^; 42 slices). The functional slices were oriented in transverse plane and were angled to be parallel to the AC-PC line, with an inline retrospective motion correction algorithm during EPI image acquisition.

### fMRI preprocessing

Data were preprocessed using the CONN-fMRI functional connectivity toolbox (www.nitrc.org/projects/conn)^43^. The preprocessing involved realignment, correction for motion artifacts with the *Artifact Detection Tools* (ART), high pass filtering (> 0.008 Hz), and co-registration to the corresponding anatomical image. Outlier detection was conducted by scanning the global BOLD signal and removing volumes associated to signal changes above 4 standard deviations, or framewise displacements above 0.5 mm. After anatomical image segmentation, functional images were normalized to *Montreal Neurological Institute* stereotaxic space and were smoothed with an 8-mm 3D isotropic Gaussian kernel. The anatomical component-based noise correction method (CompCor)^44^ was used to remove confounding effects, such as the physiological noise caused by the white matter and cerebrospinal fluid, from the BOLD timeseries^45^.

### fMRI analyses

For each participant, a general Linear Model (GLM) was mapped using SPM12 (https://www.fil.ion.ucl.ac.uk/spm/software/spm12/)^46^ in Matlab version 2020a. The experimental conditions of Self (adjectives relating to the participant), other (adjectives relating to another person) and Control (adjectives presented in uppercase or lowercase) were convolved to a canonical hemodynamic response function. Temporal correlations were accounted for by the “FAST” model^47^. Our primary contrast of interest was defined as (Other—Self) and isolates the differentiation between others and the self. We also implemented two other contrasts: the (Other—Control) and (Self—control) contrasts, which isolate other-related and self-related processing, respectively.

Between-group differences were evaluated using a full ANOVA F-test comparing the three groups for each of the three contrasts of interest (peak threshold: z > 3.1), using subject as a random effect, and pair-wise t-tests were subsequently performed. The Marsbar toolbox^48^ was used to extract the estimated parameters of the regressors in the GLM model (beta weights). The statistical threshold was determined using a Monte Carlo simulation^49^, a widely used approach that takes into account spatial smoothing and allows for less Type II errors and increased sensitivity in studies with moderate sample sizes. For the full ANOVA, after 10,000 simulations, a cluster size of 41 resampled voxels was indicated to correct for multiple comparisons at p < 0.05.

Voxel-wise seed-based functional connectivity analyses were performed in CONN using generalized psycho-physiological interaction (gPPI) analyses. The gPPI model generated a regressor of each condition (Self, Other, and Control), as this approach has been shown to generate a more accurate model of the interaction between conditions and neural activity^50^. The model contained a psychological regressor for each condition, a physiological regressor, and an interaction term. The psychophysiological interaction term was then modelled against the time course for all brain voxels, testing for significant connectivity effects. The seed region was defined based on the results of the activation analyses. Between-group differences were evaluated using a full analysis of variance (ANOVA) for each of the contrasts of interest, and the same peak and cluster thresholds as those used for the brain activity analyses. Beta weights were extracted for all significant findings.

### Statistical analyses

Between-group differences in continuous behavioral data (age, substance use and cognitive scores) were examined with ANOVAs. Post-hoc analyses were performed using Tukey’s Honestly Significant Difference test. For dichotomous data (sex and handedness), χ^2^-tests and pairwise comparisons were performed. Significance was set at p < 0.05. Analyses of covariance (ANCOVAs) assessed potential effects of clinical and behavioral variables on the between-group differences in brain activity. To test the relationship between behavioral variables (e.g. substance use and cognitive scores) and activation and connectivity strength estimates, bivariate Pearson correlation analyses were performed across and within groups (p ≤ 0.05, Bonferroni corrected).

## Results

### Demographic and behavioral variables

Demographic variables for each of the three trajectories are reported in Table 1. No significant group differences were detected for age, sex, or handedness, nor were there group differences in theory of mind scores. PLE-1 participants did, however, report higher past year alcohol use but no difference in tobacco and cannabis use. Finally, the answers to the self-other fMRI task were compiled to show the percentage of attribution of positive and negative traits to each of the “self’ and “other” conditions. There were no differences between groups in mean responses on the trait-judgement task administered in the scanner (Table 2).

**Table 1:** Demographic characteristics of the study sample.

	PLE-0 (N = 41)	PLE-1 (N = 19)	PLE-2 (N = 26)	Statistics
**Demographic**				
Age (SE)	17.22 (0.11)	17.37 (0.19)	17.31 (0.17)	F = 0.25; p = 0.78
Sex, % male	46.3	42.11	53.84	χ^2^ = 0.66; p = 0.72
Handedness, % right-handed	97.56	94.74	96.15	χ^2^ = 0.851; p = 0.85
**Substance use (12 m)**				
Cannabis use level (SE)	0.87 (0.21)	1.5 (0.41)	1.3 (0.18)	F = 1.18; p = 0.313
Alcohol use level (SE)	2 (0.16)	2.61* (0.20)	1.56* (0.23)	F = 5.68; p = 0.005
Tobacco use level (SE)	0.76 (0.25)	1.39 (0.40)	0.74 (0.36)	F = 1.05; p = 0.356
**Theory of Mind task results**				
Second-order mentalizing (SD)	88.25% (6.91)	87.78% (7.75)	88.23% (5.62)	F = 0.03; p = 0.97
Non-social reasoning control (SD)	92.59% (8.40)	95.10% (8.87)	95.33% (6.84)	F = 1.05; p = 0.35
First-order inference control (SD)	96.76% (9.61)	100% (0)	97.33% (9.23)	F = 0.89; p = 0.42
Attention and memory control (SD)	99.43% (1.30)	95.88% (1.94)	95.33% (2.44)	F = 1.38; p = 0.26
False positive control (SD)	86.11% (22.71)	91.17% (19.65)	82% (28.43)	F = 0.74; p = 0.48

SE = Standard Error; SD = Standard Deviation; Substance use rated on a 6-point scale; The theory of mind task looked at stories of false belief which served as a measure of second-order mentalizing, control items tested non-social reasoning, first-order inference, questions to control for attention and memory effect, and items to control for false-positives. Mean percentage of correct answers in each category.

PLE-0 = control trajectory; PLE-1 = decreasing trajectory; PLE-2 = increasing trajectory.

*Significant difference.

**Table 2:** Self-other task cognitive trait attribution results.

	PLE-0 (N = 41)	PLE-1 (N = 19*)	PLE-2 (N = 26*)
Self-positive (SD)	78.70% (22.00)	81.25% (13.46)	83.92% (10.59)
Self-negative (SD)	28.21% (18.69)	26.04% (17.71)	21.97% (14.44)
Other-positive (SD)	70.02% (20.87)	75.96% (17.06)	78.32% (14.17)
Other-negative (SD)	21.15% (16.87)	21.35% (15.21)	23.11% (10.89)

SD = Standard Deviation; Mean percentage of “yes” responses in each category. No significant group differences were found.

PLE-0 = control trajectory; PLE-1 = decreasing trajectory; PLE-2 = increasing trajectory.

*Missing data for some participants in decreasing and increasing trajectory groups.

### fMRI results

**Figure 2:**
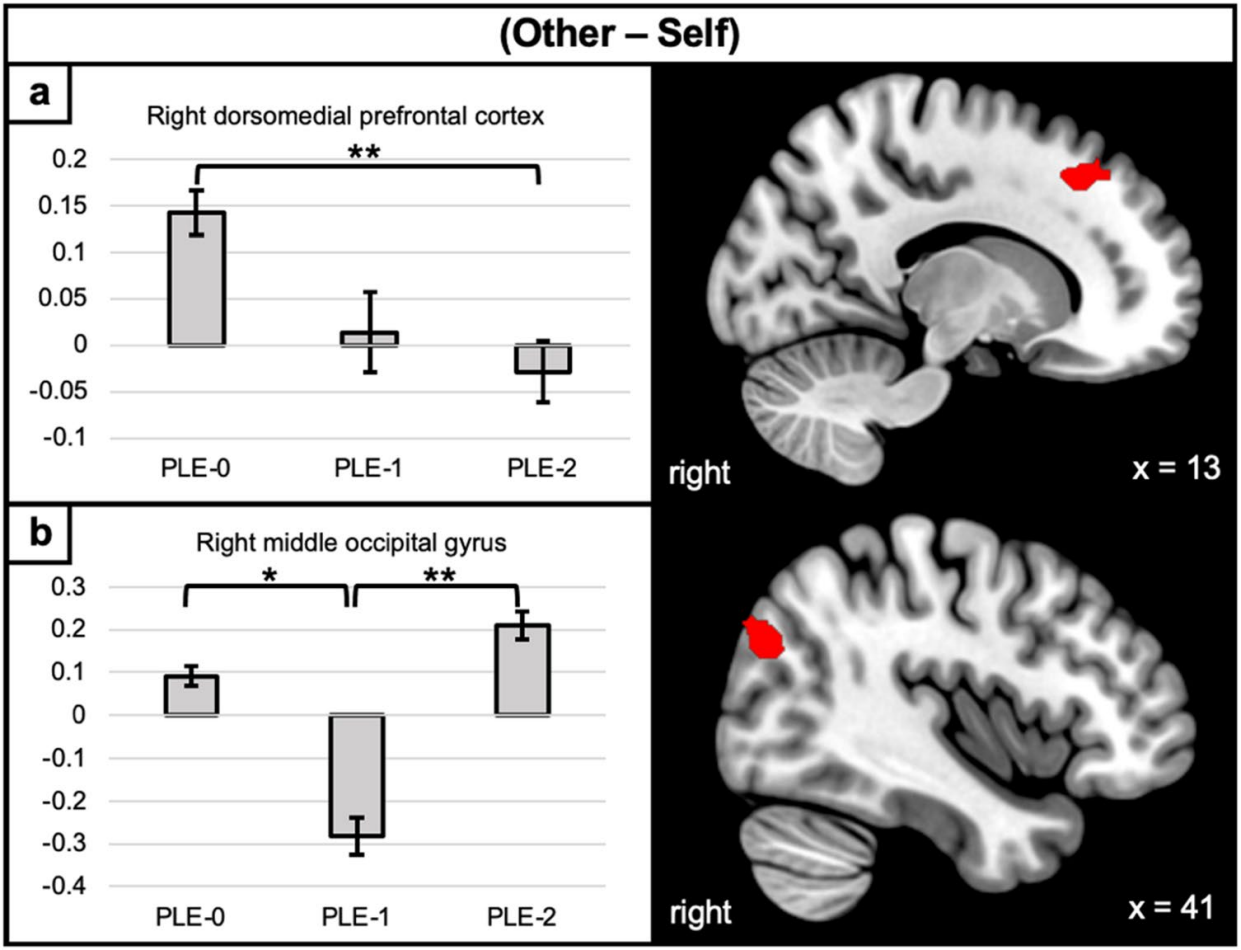
between-group differences in activation levels during the (Other—Self) contrast. Activation maps the clusters of between-group differences during the (Other—Self) contrast isolating the differentiation and integration of other- and self- related information. Error bars correspond to the standard error. PLE-0 = control trajectory; PLE-1 = decreasing trajectory; PLE-2 = increasing trajectory. *p < 0.005. **p < 0.001.

For all contrasts, results are reported in Table 3. Within-group results are also reported in the supplementary information and show task-relevant activations. Group differences were found in the (Other—Self) and (Other—Control) contrasts. In the (Other—Self) contrast (Fig. 2), the increasing PLE-2 trajectory displayed decreased activation of the right dorsomedial PFC, relative to the control PLE-0 trajectory. The decreasing PLE-1 trajectory displayed decreased activation of the right middle occipital gyrus, relative to control PLE-0 individuals.

**Table 3:** Peak coordinates of areas with between-group differences in activation levels.

Contrast	Region	Peak coordinates			F-value	Cluster size
		x	y	z		
(Self—Other)	Right dorsomedial prefrontal cortex	12	34	44	9.38	118
	Right middle occipital gyrus	40	− 74	32	8.37	99
(Other—Control)	Left superior temporal gyrus	− 50	0	− 10	9.83	103
	Right inferior frontal gyrus (opercular)	62	18	16	9.62	94
	Right inferior frontal gyrus (pars orbitalis)	48	30	− 6	9.54	389

This table shows the results of the whole-brain activation analysis for the different contrasts, with the corresponding peak region names, their MNI coordinates, peak F value, and cluster size.

**Figure 3:**
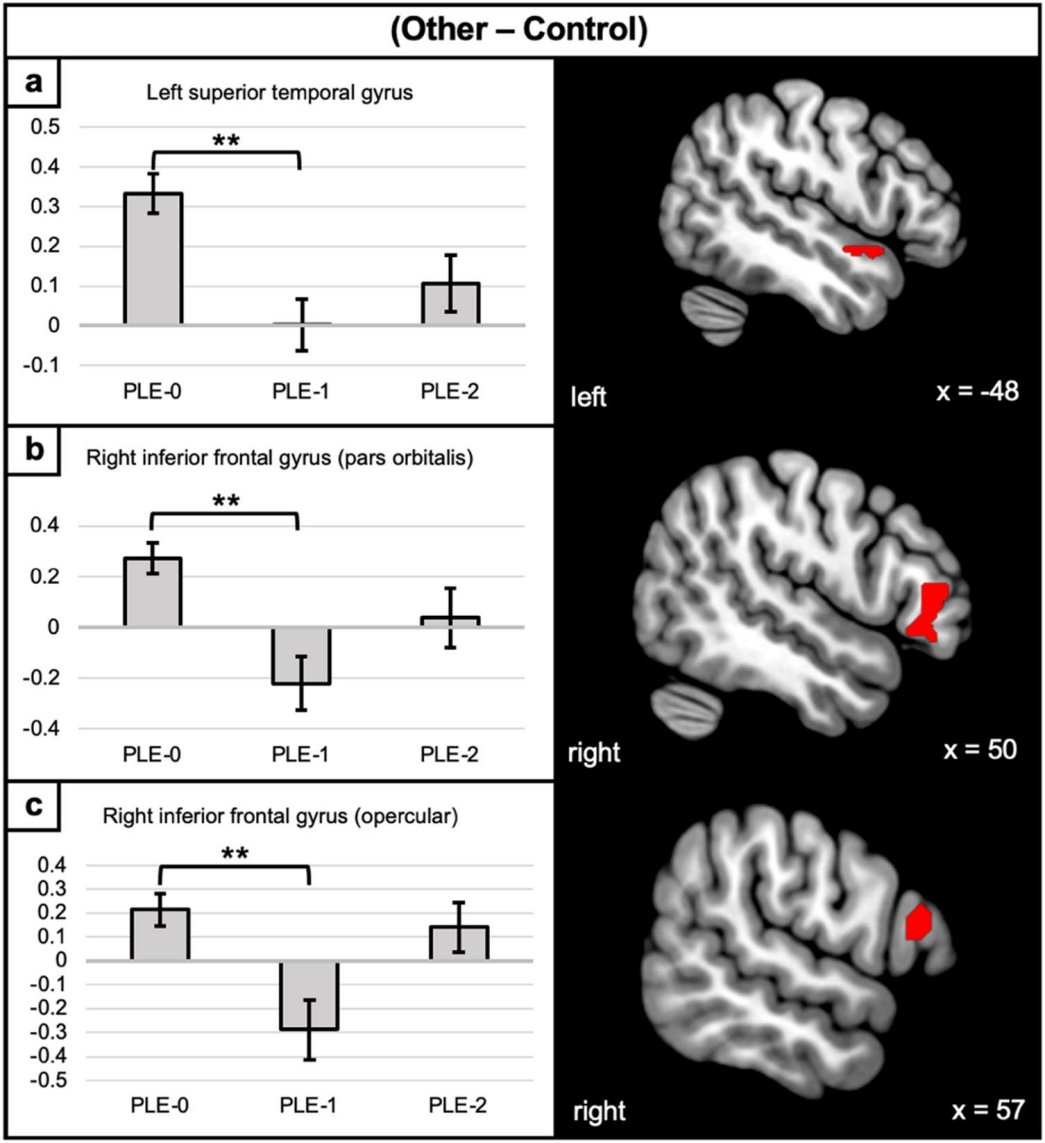
Between-group differences in activation levels during the (Other—Control) contrast. Activation maps of the clusters of between-group differences during the (Other—Control) contrast isolating other-related processing. Error bars correspond to the standard error. PLE-0 = control trajectory; PLE-1 = decreasing trajectory; PLE-2 = increasing trajectory. **p < 0.001.

In the (Other—Control) contrast (Fig. 3), the PLE-1 trajectory displayed decreased activation of the left superior temporal gyrus and the right inferior frontal gyrus (pars orbitalis and pars opercularis) compared to the control trajectory PLE-0. All results remained significant after controlling for alcohol use.

For all activation estimates, post-hoc analyses of covariance were conducted to assess the effects of sex and age on group differences, and all the above-mentioned results remains significant.

**Figure 4:**
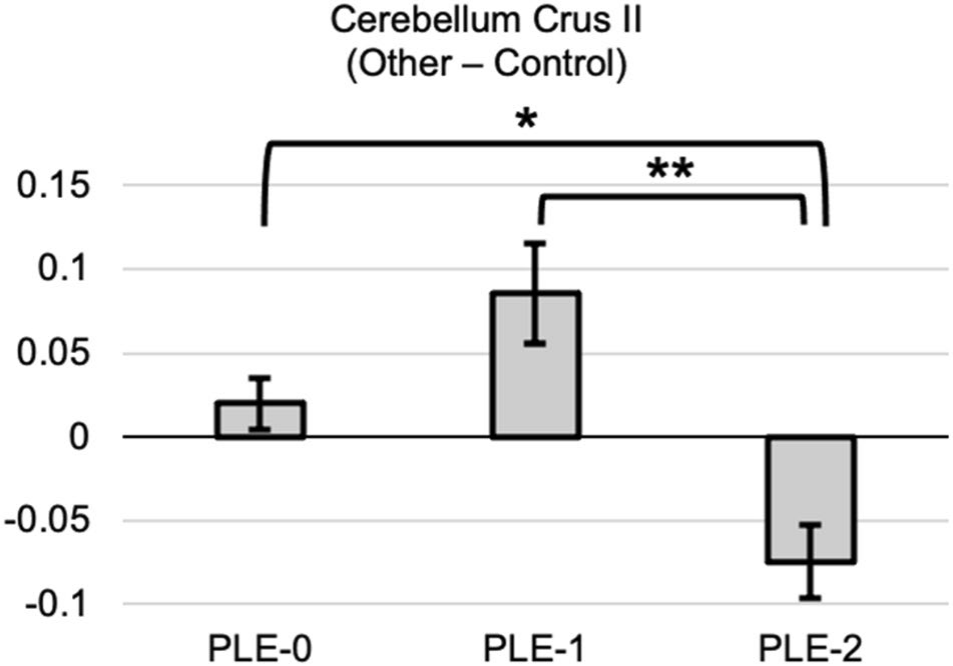
seed-based functional connectivity in the (Other—Control) contrast. Bar graphs showing the between-group functional connectivity results (PLE-0 = control trajectory; PLE-1 = decreasing trajectory; PLE-2 = increasing trajectory). The bar graph shows the beta connectivity scores for the (Other—Control) contrast. Error bars correspond to the standard error. *p < 0.005. **p < 0.001.

Seed-based connectivity analyses were conducted using the dorsomedial PFC coordinates identified in the activation analyses. This seed was selected due to its significant role in self-other processing and its coordinates are consistent with the literature’s definition of the dorsomedial PFC, as determined by a recent meta-analysis of fMRI studies^24^. Comparatively, the other regions identified in the activation analyses are not assumed to play a direct role in self-other processing. Results are reported in Table 4. In the (Other—Control) contrast (Fig. 4), the increasing PLE-2 trajectory displayed decreased functional connectivity between the right dorsomedial PFC and the Crus II region of the right cerebellum, compared to the control PLE-0 and decreasing PLE-1 trajectories.

**Table 4:** between-group connectivity differences.

Contrast	Seed	Region	Peak coordinates			Cluster size
			x	y	z	
(Other—Control)	Right dorsomedial prefrontal cortex	Right cerebellum Crus II	12	− 86	− 32	71

This table shows the significant results of the seed-to-voxel functional connectivity analysis. The selected seed is presented with the corresponding target voxel, its MNI coordinates, and cluster size.

### Correlation analyses

Past year cannabis use level was found to be positively correlated (r = 0.339; p = 0.002) with the activation strength of the right middle occipital gyrus in the (Self—Other) contrast. No other behavioral variable was significantly correlated with fMRI results.

## Discussion

This study showed that PLE trajectories that are associated with elevated positive vs. negative symptoms exhibit distinct neural alterations during self-other processing. The increasing PLE-2 trajectory (positive symptom risk) was associated with decreased activation of the right dorsomedial PFC during other-self distinction. The decreasing trajectory PLE-1 (negative symptom risk) was associated with decreased activation of the right middle occipital gyrus during self-other distinction and decreased activation of the left superior temporal gyrus and right IFG during other-processing.

As hypothesized, we found neural alterations during self-other differentiation in the increasing trajectory which we previously showed to be at greater risk for developing positive symptoms of psychosis. The dorsomedial PFC is activated in healthy volunteers during self-processing and is involved in the integration and separation of self- vs. other- related information^51,52^. The self-other processing related hypoactivations seen here in the increasing trajectory have also been observed in people with schizophrenia^28,53,54^, which, interestingly, were also not shown to correlate with behavioural measures of self- and other- processing^53^. Despite heterogeneity in findings across studies^54^, one of the most consistent findings during self-other processing in schizophrenia is the reduced activity observed in a large cluster encompassing the dorsomedial PFC and the dorsal anterior cingulate cortex^28^. It will be important to develop more relevant behavioural tasks of self-other processing that better dissociate high risk individual, and which better capture what is being measured in fMRI tasks. For example, perhaps such tasks will be more sensitive when focusing on physiologic or subjective outcomes, rather than behavioural.

The increasing trajectory also displayed hypoconnectivity between the dorsomedial PFC and the Crus II region of the cerebellum during other-related processing. This area of the cerebellum is thought to be involved in mentalizing^55^, and decreased prefrontal-cerebellar connectivity has been found to correlate with self-disturbances in psychosis and in ultra-high risk individuals^56^. It has been proposed that impairments in integrating or differentiating self- and other-related information may underlie the emergence of other symptoms of psychosis such as hallucinations^57^. According to this model, hallucinations would arise from a blurred distinction between the self and other, such that internal speech can be wrongfully attributed to external sources. Our findings bolster this view, suggesting further that deficits in self-other differentiation and integration affect the early development of psychosis, specifically within the increasing trajectory.

With respect to the decreasing PLE trajectory, which we previously showed to predict risk for negative symptoms^11^, the current study showed that this group exhibited a more generalized profile of neural alterations during other-related processing, including hypoactivation of the right IFG, left superior temporal cortex and middle occipital gyrus. None of these regions are thought to play a critical role in self-other processing. The right IFG is usually recruited in cognitive tasks requiring the detection of novel or task-relevant cues, indicating its role in attentional control and/or attentional salience^58^. The IFG can also be activated during tasks requiring semantic encoding during in trait judgements regardless of the condition (self or other)^59^, yet not in control judgements (e.g., uppercase vs. lowercase characters)^41,60^. However, these results have not been consistently replicated. Taken together, these results suggest that the decreasing PLE trajectory displays alterations in regions more implicated in attentional and cognitive control than during self-other processing.

The decreasing trajectory also displayed a hypoactivation of the left superior temporal gyrus, which has been shown to exhibit altered connectivity during other-processing in individuals with schizophrenia^61^. While the anterior part of the left superior temporal gyrus is involved socio-emotion processing^62^, its precise role in self-other processing remains uncertain. In the (Other—Self) contrast, the decreasing trajectory displayed decreased activation of the right middle occipital gyrus, which is known to be involved prominently in visual information processing^63^. The middle occipital gyrus was found to exhibit structural changes associated with poor insight in psychosis^64^, however the meaning of this association remains elusive. In our sample, the activation level of the middle occipital gyrus was positively correlated with past year cannabis use across groups, which is consistent with a meta-analysis that found that cannabis use was associated with altered activation of this region^65^. These findings suggest that the decreasing trajectory is associated with a range of alterations in areas that are not specific to self-other processing, but are more generally involved in attention, basic information processing, and potentially a pattern of substance use that is highly comorbid with psychosis, which may explain the increased non-specific negative-like symptoms (avolition, social withdrawal, affective flattening) in this group.

While the present study offers novel information on a key pre-clinical population, it presents some limitations. Although studies of self-other processing have used similar sample sizes in schizophrenia^29,32,66^, the number of participants in this study was limited and unbalanced due to the low prevalence of the atypical trajectories in the general population cohort, with only 8% of youths on either decreasing and increasing trajectories, and the difficulty in recruiting these participants. Future studies would benefit from increased participants as the smaller sample size could account for the absence of significant differences on the behavioural measures. It is worth noting that while the decreasing and increasing trajectories displayed group differences at the level of positive and negative symptoms^11^, no correlations between symptoms and activation and connectivity measures were found at the individual level. This may be due to the subclinical nature of symptoms. Similarly, no behavioral differences were found in the Theory of Mind task between groups. This may be because this task isolates different constructs than the fMRI task, whereby it not only requires the participant to think about others, but it also involves the integration of various social cues (intention, context, sarcasm etc.). The inclusion of a scale on self-disturbances, such as the Examination of Anomalous Self-Experience (EASE) scale^67^, may have produced better results. It is also possible that the Theory of Mind task—which has been validated in adult patients—is not adapted to discern differences in this at-risk population. Finally, this study did not include additional measures related to self-other processing, and future studies should implement multimodal testing that evaluates different aspects of social cognition to evaluate whether these neural alterations translate into impairments in social functioning. These features noted, our findings highlight the relevance of assessing developmental trajectories of PLEs when studying the early neural markers of psychosis.

In conclusion, the current study showed that youths with atypical PLE trajectories display different neural alterations during self-other processing. The main finding of impaired dorsomedial PFC activations in the increasing trajectory is similar to results reported in schizophrenia. Considering that the dorsomedial PFC plays a crucial role in self-other differentiation and that this trajectory predicts susceptibility to more positive psychotic-like symptoms in young adulthood, the altered dorsomedial PFC activation during self-other processing should be further investigated as a biomarker of psychosis risk. In comparison, the decreasing trajectory (at risk of more negative symptoms) displayed hypoactivations in different areas that are involved in various functions, indicating a less specific profile of neural changes. Confirmatory studies using predefined regions of interest should be conducted on these trajectories to further validate these findings. Future longitudinal studies following atypical trajectories of psychotic experiences should assess the evolution of these alterations and their capacity to predict clinical symptom emergence in at-risk youth.

## Supplementary Information

Supplementary Information.

## Data Availability

The data are not publicly available as they contain information that could compromise research participant privacy/consent. The data that support the findings of this study are available upon reasonable request from the corresponding author. S.P. but are only redistributable to researchers engaged in IRB approved research collaborations.
