# Supplementary material for: A functional neuroimaging study of self-other processing alterations in atypical developmental trajectories of psychotic-like experiences
Source: Sci Rep. 2022 Sep 29;12:16324. doi: 10.1038/s41598-022-20129-3 (PMC9522794; doi:10.1038/s41598-022-20129-3)
Supplement: Supplementary file 1 — Supplementary Information. [file 41598_2022_20129_MOESM1_ESM.docx]

**Supplementary table 1: within-group PLE-0 activation maps in the various contrasts of interests**

| **Contrast** | **Region** | **Coordinates** | | | **T-value** | **Cluster size** |
| --- | --- | --- | --- | --- | --- | --- |
|  |  | **x** | **y** | **z** |  |  |
| **Other -Self** | right precuneus | 0 | -62 | 40 | 7.83 | 3216 |
|  | right ventral posterior cingulate cortex | 6 | -54 | 26 | 7.15 |  |
|  | left ventral posterior cingulate cortex | -4 | -52 | 26 | 5.99 |  |
|  | right angular gyrus | 38 | -58 | 34 | 6.95 | 1531 |
|  | right angular gyrus | 56 | -68 | 28 | 6.72 |  |
|  | right angular gyrus | 40 | -62 | 42 | 6.07 |  |
|  | right superior frontal gyrus | 14 | 36 | 46 | 6.65 | 1077 |
|  | right middle frontal gyrus | 22 | 28 | 46 | 6.34 |  |
|  | right middle frontal gyrus | 30 | 10 | 38 | 4.67 |  |
|  | left angular gyrus | -38 | -62 | 32 | 6.62 | 2276 |
|  | left angular gyrus | -42 | -70 | 48 | 5.53 |  |
|  | left angular gyrus | -54 | -68 | 32 | 5.31 |  |
|  | left medial temporal gyrus | -58 | -10 | -16 | 6.24 | 1059 |
|  | left medial temporal gyrus | -48 | -24 | -12 | 4.48 |  |
|  | left medial temporal gyrus | -66 | -36 | -8 | 4.28 |  |
|  | right medial temporal gyrus | 64 | -8 | -20 | 6.03 | 276 |
|  | right anterior prefrontal cortex | 8 | 56 | -10 | 6.02 | 432 |
|  | left middle frontal gyrus | -28 | 14 | 52 | 4.96 | 1071 |
|  | left superior frontal gyrus | -16 | 16 | 46 | 4.35 |  |
|  | left middle frontal gyrus | -16 | 32 | 44 | 4.17 |  |
|  | left posterior cerebellum | -10 | -86 | -38 | 4.67 | 110 |
|  | left posterior cerebellum | -4 | -82 | -34 | 4.53 |  |
|  | right posterior cerebellum | 12 | -86 | -38 | 3.52 |  |
|  | left medial frontal gyrus | -8 | -14 | 52 | 4.47 | 50 |
|  | right dorsolateral prefrontal cortex | 54 | 28 | 32 | 4.42 | 203 |
|  | right dorsolateral prefrontal cortex | 38 | 22 | 30 | 3.71 |  |
|  | left precentral gyrus | -28 | -26 | 54 | 4.04 | 107 |
|  | left precentral gyrus | -32 | -28 | 64 | 3.76 |  |
|  | left precuneus | -4 | -40 | 72 | 3.67 | 86 |
| **Other - Control** | left superior frontal gyrus | -6 | 18 | 64 | 11.88 | 45677 |
|  | left medial frontal gyrus | -4 | 18 | 48 | 11.43 |  |
|  | right posterior cerebellum | 26 | -86 | -36 | 10.82 |  |
|  | right cuneus | 26 | -98 | -6 | 7.73 | 699 |
|  | right middle occipital gyrus | 36 | -94 | -4 | 7.38 |  |
|  | right middle occipital gyrus | 36 | -94 | 10 | 4.89 |  |
|  | right medial temporal gyrus | 64 | -6 | -18 | 5.1 | 257 |
|  | right superior temporal gyrus | 52 | -12 | -16 | 4.1 |  |
|  | right angular gyrus | 58 | -62 | 36 | 4.92 | 49 |
|  | left secondary visual | -12 | -98 | 26 | 4.86 | 43 |
|  | left cuneus | -8 | -90 | 36 | 3.76 |  |
|  | right precuneus | 18 | -42 | 6 | 4.31 | 77 |

**Supplementary table 2: within-group PLE-1 activation maps in the various contrasts of interests**

| **Contrast** | **Region** | **Coordinates** | | | **T-value** | **Cluster size** |
| --- | --- | --- | --- | --- | --- | --- |
|  |  | **x** | **y** | **z** |  |  |
| **Other - Self** | left angular gyrus | -46 | -66 | 48 | 4.9 | 247 |
|  | left angular gyrus | -48 | -66 | 34 | 3.67 |  |
|  | right ventral posterior cingulate cortex | 4 | -52 | 28 | 4.26 | 344 |
|  | right precuneus | 0 | -58 | 36 | 3.68 |  |
| **Other - Control** | left dorsolateral prefrontal cortex | -10 | 54 | 36 | 10.77 | 6907 |
|  | right anterior prefrontal cortex | 0 | 58 | 16 | 8.94 |  |
|  | left dorsolateral prefrontal cortex | -22 | 52 | 38 | 7.71 |  |
|  | left thalamus | -6 | -28 | 4 | 10.22 | 6998 |
|  | right thalamus | 2 | -2 | 6 | 9.63 |  |
|  | right lingual gyrus | 4 | -32 | -6 | 8.96 |  |
|  | right posterior cerebellum | 28 | -78 | -28 | 7.76 | 695 |
|  | right posterior cerebellum | 22 | -88 | -28 | 6.61 |  |
|  | right posterior cerebellum | 42 | -78 | -32 | 5.74 |  |
|  | left medial temporal gyrus | -58 | -6 | -18 | 6.1 | 244 |
|  | left medial temporal gyrus | -50 | -10 | -18 | 5.57 |  |
|  | right cuneus | 20 | -104 | 6 | 6.03 | 57 |
|  | right middle occipital gyrus | 40 | -90 | 8 | 4.73 |  |
|  | right middle occipital gyrus | 30 | -100 | 6 | 4.33 |  |
|  | left ventral posterior cingulate cortex | -26 | -62 | 6 | 5.63 | 137 |
|  | left cuneus | -24 | -72 | 6 | 4.48 |  |
|  | left precuneus | -18 | -50 | 6 | 4.26 |  |
|  | left middle temporal gyrus | -48 | -66 | 28 | 5.43 | 143 |
|  | left angular gyrus | -50 | -74 | 36 | 4.33 |  |
|  | right caudate nucleus | 12 | -34 | 16 | 5.06 | 62 |
|  | right caudate nucleus | 20 | -34 | 20 | 4.67 |  |
|  | right cuneus | -16 | -104 | -8 | 4.48 | 43 |
|  | right posterior cerebellum | 34 | -62 | -26 | 4.43 | 47 |

**Supplementary table 3: within-group PLE-2 activation maps in the various contrasts of interests**

| **Contrast** | **Region** | **Coordinates** | | | **T-value** | **Cluster size** |
| --- | --- | --- | --- | --- | --- | --- |
|  |  | **x** | **y** | **z** |  |  |
| **Other - Self** | right precuneus | 0 | -58 | 30 | 6.01 | 1548 |
|  | right ventral posterior cingulate cortex | 8 | -52 | 28 | 5.53 |  |
|  | left precuneus | -12 | -52 | 30 | 5.44 |  |
|  | right insula | 44 | -14 | 16 | 5.71 | 243 |
|  | right precentral gyrus | 56 | -8 | 10 | 4 |  |
|  | right insula | 40 | -6 | 12 | 4 |  |
|  | left middle temporal gyrus | -34 | -62 | 22 | 5.42 | 611 |
|  | left angular gyrus | -38 | -66 | 32 | 5.2 |  |
|  | left angular gyrus | -38 | -64 | 42 | 4.37 |  |
|  | right postcentral gyrus | 70 | -18 | 24 | 4.39 | 113 |
|  | right postcentral gyrus | 66 | -22 | 38 | 3.92 |  |
|  | right middle temporal gyrus | 52 | -70 | 18 | 4,34 | 93 |
|  | right superior temporal gyrus | 60 | -62 | 18 | 3.93 |  |
| **Other - Control** | left ventral posterior cingulate gyrus | -4 | -50 | 28 | 8.81 | 13152 |
|  | right ventral posterior cingulate gyrus | 6 | -48 | 28 | 7.53 |  |
|  | left temporal pole | -48 | 8 | -26 | 7.52 |  |
|  | right posterior cerebellum | 30 | -78 | -34 | 6.51 | 469 |
|  | right posterior cerebellum | 16 | -88 | -30 | 4.84 |  |
|  | right posterior cerebellum | 8 | -84 | -24 | 3.9 |  |
|  | right middle occipital gyrus | 30 | -98 | -2 | 6.28 | 186 |
|  | right middle occipital gyrus | 38 | -94 | -4 | 5.22 |  |
|  | right secondary visual cortex | 24 | -96 | 18 | 4.53 |  |
|  | left angular gyrus | -36 | -52 | 22 | 6.01 | 300 |
|  | left angular gyrus | -44 | -62 | 26 | 4.65 |  |
|  | right temporal pole | 52 | 12 | -28 | 5.79 | 171 |
|  | right middle temporal gyrus | 60 | -2 | -28 | 4.29 |  |
|  | left posterior cerebellum | -4 | -38 | -42 | 5.54 | 97 |
|  | right posterior cerebellum | 2 | -54 | -40 | 4.15 |  |
|  | left posterior cerebellum | -6 | -56 | -40 | 4.13 |  |
|  | left cuneus | -10 | -98 | 18 | 5.07 | 80 |
|  | left cuneus | -6 | -94 | 30 | 4.91 |  |
|  | left cuneus | -4 | -102 | 8 | 3.49 |  |
|  | left cuneus | -26 | -98 | -4 | 3.962 | 327 |
|  | left middle occipital gyrus | -32 | -94 | 6 | 3.96 |  |
|  | left cuneus | -14 | -104 | 2 | 3.66 |  |
|  | left posterior cerebellum | -30 | -80 | -38 | 4.4 | 53 |
|  | left middle frontal gyrus | -36 | 14 | 52 | 4.32 | 91 |
|  | left medial temporal gyrus | -50 | -40 | 2 | 3.99 | 57 |
